# Effects of a Mindfulness-Based Parental Reflection Intervention on Pregnancy-Related Distress: A Pilot Study

**DOI:** 10.1089/whr.2022.0090

**Published:** 2023-02-09

**Authors:** Lucia Ciciolla, Samantha Addante, Karina M. Shreffler, Julie M. Croff

**Affiliations:** ^1^ Department of Psychology, Oklahoma State University, Stillwater, Oklahoma, USA.; ^2^ College of Nursing, University of Oklahoma Health Sciences Center, Oklahoma City, Oklahoma, USA.; ^3^ Center for Health Sciences, Oklahoma State University, Stillwater, Oklahoma, USA.

**Keywords:** pregnancy, distress, maternal mental health, mindfulness, intervention

## Abstract

**Background:**

Mindfulness-based interventions have been shown to be efficacious for reducing psychological distress and mental health symptoms and promoting well-being, including during pregnancy and postpartum. There is promising, though limited, evidence showing that interventions that focus on improving the mother-infant relationship are associated with improvements in both the mother-infant relationship and maternal mental health symptoms. The current study examines the effects of a prenatal mindfulness-based, reflective intervention designed to enhance maternal-fetal bonding on pregnancy-related distress and prenatal depressive symptoms.

**Methods:**

Out of a larger sample of 130 pregnant women in their second trimester, 15 women were recruited to participate in a 2-week long mindfulness-based, reflective intervention with daily short (<5-minute) activities. Multiple linear regression analyses were conducted to examine associations between the intervention and pregnancy-related distress and depression during the third trimester of pregnancy, controlling for race, age, education, union status, and first trimester depressive symptoms.

**Results:**

Results indicate that women who participated in the intervention during their second trimester reported lower pregnancy-related distress in their third trimester but no differences in depressive symptoms.

**Conclusions:**

A brief, mindfulness-based intervention delivered during pregnancy via cellphone texts can be a useful tool to reduce maternal distress related to pregnancy. Additional reflective exercises that address mood and global stress, as well as increasing the amount and/or frequency of the intervention, may be important for promoting maternal mental health more globally.

## Introduction

Psychological distress during pregnancy, including symptoms of depression and anxiety, has been associated with low levels of maternal-fetal bonding^1,2^ and impaired infant development.^3^ Symptoms of psychological distress, including feeling overwhelmed, excessive worry, and feelings of helplessness or hopelessness, may limit mothers' cognitive and emotional ability to bond during pregnancy^4^ and postpartum.^5–7^

Stress during pregnancy has been found to predict adverse birth outcomes,^8,9^ neonatal cortisol levels,^10^ and postpartum mental health.^10^ Improving maternal prenatal mental health, therefore, has critical implications for supporting infant social, emotional, and cognitive development.

Mindfulness-based interventions have been shown to be efficacious for reducing psychological distress and mental health symptoms and promoting well-being,^11^ including during pregnancy and postpartum.^12–15^ Mindfulness-based interventions promote attentional awareness of one's internal mental processes in the present moment with a focus on the principles of nonjudgment, compassion, self-regulation, and cultivating emotional awareness^16^ and often include meditation or other contemplative practice.

However, although mindfulness ability has been linked to maternal-fetal bonding,^17^ interventions that focus solely on addressing maternal symptomology but do not address the mother-infant relationship are insufficient for supporting and improving disruptions to the mother-infant relationship.^18–21^

There is promising, though limited, evidence that interventions that focus on enhancing the mother-infant relationship also improve maternal mental health symptoms.^22–24^ Interventions for improving maternal-fetal bonding inherently focus on the emotional and cognitive skills that support reflective functioning or mentalizing, including forming mental representations of the fetus, perspective-taking, and developing empathy.^25^

Mentalization and mindfulness act to direct one's attention to one's own experience and emphasize the integration of cognitive and affective aspects of mental states. However, the timescale is different for each construct wherein mindfulness orients to the present and mentalization focuses on an anticipated future based on present events. Moreover, mindfulness emphasizes acceptance of one's inner experience and mentalization emphasizes construction of a representation and meaning related to one's inner experiences.^26^

When used in combination, mindfulness and mentalization serve to orient a person to current circumstances and envision a future based on current events, without heightened emotionality or worry associated with past events. Many parenting interventions incorporate aspects of both mentalization and mindfulness, which may help to explain why they serve to improve maternal mental health.^22,27^

However, these intervention studies are limited to the postpartum period, and although there are reports on prenatal interventions that show improvement in maternal-fetal bonding specifically,^28,29^ a few studies to date have examined the effect of a prenatal mindfulness-based intervention on maternal distress or mental health symptoms during pregnancy.^28^

The current pilot study examines the effects of a mindfulness-based, reflective prenatal intervention designed to enhance maternal-fetal bonding and maternal prenatal mental health; specifically, we examine the impact of the intervention delivered in the second trimester of pregnancy on pregnancy-related distress and depressive symptoms in the third trimester. It is expected that mothers who received the intervention would report lower levels of pregnancy-related distress and depressive symptoms compared with controls.

## Methods

### Sample

The sample for the present study consists of 130 pregnant women (16–38 years of age) who responded to surveys during their first through third trimesters of pregnancy and had data on pregnancy-related distress and depressive symptoms. Following Oklahoma State University Institutional Review Board full board approval (protocol no. HE1570), participants were recruited from two prenatal clinics serving high proportions of Medicaid patients.

Participants completed online surveys about their pregnancy experiences and perceptions, including questions about their mental health and sociodemographic information. Women who participated in the first assessment of the study were invited to participate in the Babies and Moms connected by Love, Openness, and Opportunity (BLOOM) intervention if they were in their second trimester during the 2 months that BLOOM was piloted during the larger study.

The current study focuses on those who received the BLOOM mindfulness-based reflective exercises designed to enhance feelings of maternal-fetal bonding (*n* = 15) compared with 115 women who did not receive the mindfulness-based intervention. There were initially two interventions tested in the pilot study, but the other intervention technique (using a fetal doppler to listen to the fetal heartbeat, *n* = 12) was not found to have an independent effect in analyses.

For the current study, participants who only received the doppler intervention (*n* = 7) were included in the control group (*i.e.*, no mindfulness-based intervention), whereas participants who received the doppler plus the mindfulness-based reflective exercises (*n* = 5) were included in the intervention group. Comparisons of participants who received both the doppler and the mindfulness-based reflective exercises to the exercise-only group showed no differences in pregnancy-related distress or prenatal depression.

The 2-week BLOOM intervention was conducted during the second trimester. The participants in the intervention group were sent daily activities that incorporated both mindfulness and reflective mentalization via text messages (mid-evening). The activities were designed to be short in length, averaging 5 minutes or less per exercise. The mindfulness-based reflective exercises included deep breathing, meditation, prenatal massage, responding to kicks, nursery rhymes, telling the baby about a cherished person in their life, planning an activity with the baby, and reading a story to the baby.

### Measures

The primary independent variable for the study is the *BLOOM intervention* group (coded as 1 = received mindfulness-based reflective exercises; 0 = no intervention).

The first dependent variable for this study, *pregnancy-related distress*, was assessed using the Prenatal Distress Questionnaire (PDQ)^30^ during the third trimester of pregnancy. The PDQ is a 12-item scale that measures worries and concerns related to pregnancy regarding physical symptoms, relationships, parenting, medical problems, labor and delivery, and the health of the baby. Items were rated on a 5-point scale ranging from 0 (not at all) to 4 (extremely) and were summed to create a scale ranging from 0 to 48.

Example items include “I worry about having an unhealthy baby”; and “I am anxious about labor and delivery.” The Cronbach's alpha for the scale with this sample is 0.84, indicating good reliability. The PDQ was developed to measure pregnancy-related distress and has demonstrated reliability and convergent, concurrent, and predictive validity.^31^

The second dependent variable, *depressive symptoms*, was assessed by the Center for Epidemiological Studies-Depression (CES-D)^32^ scale during the third trimester of pregnancy. The CES-D is a 20-item scale that aims at depressive symptoms over the past 2 weeks. Items were rated on a 4-point scale ranging from 0 (rarely or none of the time) to 3 (most or almost all the time) and were summed with higher scores indicating greater depression.

A score of 16 or above indicates clinical risk for depression. The range for the third trimester assessment of the CES-D scale in the current study is 0–47, and Cronbach's alpha for this sample in the third trimester is 0.89 with this sample. The CES-D scale has demonstrated reliability and validity for use in the general population^33^ and for pregnant women.^34,35^

Several control variables previously identified as associated with prenatal distress were included in the study, including *depressive symptoms* using the CES-D scale^32^ assessed during the first trimester of pregnancy. The range for the first trimester assessment of the CES-D scale in the current study is 0–43. The Cronbach's alpha for this sample in the first trimester is 0.90, indicating excellent reliability.

Additional sociodemographic control variables include *age* as a continuous variable ranging from 16 to 38, *education* measured in years, living in a coresidential *union* with a husband or partner (1 = living in union; 0 = not living in union); and race/ethnicity coded into dummy variables for *Black, Hispanic*, and *Native* with *Non-Hispanic White* as the reference group.

### Analytic strategy

*T*-tests were used to examine differences in study variables by intervention group. Multiple linear regression analyses were conducted to estimate the association between the BLOOM intervention and pregnancy-related distress and depressive symptoms scores, controlling for race, age, education, union status, and first trimester depressive symptoms.

## Results

Descriptive statistics of the study variables are presented in Table 1 by intervention group status (no intervention vs. BLOOM intervention). *T*-test results indicate that the only variable that differed significantly by group was the dependent variable of pregnancy-related distress (*M* = 16.23, SD = 8.06 vs. *M* = 11.67, SD = 6.61; *p* = 0.03 for no intervention vs. BLOOM intervention, respectively). Sociodemographics, first trimester depressive symptoms, and third trimester depressive symptoms did not vary significantly by group.

**Table 1.** Descriptive Statistics of Study Variables

Variables	Total sample	No intervention	BLOOM intervention	T-test or chi-square
	*n* = 130	*n* = 115	*n* = 15	
	M (SD)	M (SD)	M (SD)	T or χ^2^
Pregnancy-related distress	15.7 (8.01)	16.23 (8.05)	11.67 (6.61)	−2.1^*^
T1 Depressive symptoms	14.52 (8.81)	14.49 (8.61)	14.86 (10.64)	0.15
T3 Depressive symptoms	15.38 (9.79)	15.51 (9.73)	14.47 (10.52)	−0.39
Age (years)	25.84 (5.59)	25.75 (5.67)	26.53 (5.11)	0.51
Education (years)	12.91 (2.04)	12.87 (2.03)	13.14 (2.11)	0.46
Union (living with partner)	52.3%	52.2%	53.3%	0.007
Race/ethnicity				
White	39.2%	36.5%	60%	3.97
Black	28.5%	30.4%	0.13.3%	1.91
Hispanic	13.1%	13.9%	6.7%	0.61
Native	18.5%	18.3%	20%	0.03

T1 = first trimester; T3 = third trimester; ^*^p < 0.05.

BLOOM, Babies and Moms connected by Love, Openness, and Opportunity.

Multiple linear regression analyses were conducted to examine associations between the BLOOM intervention and pregnancy-related distress and third trimester depressive symptoms controlling for age, education, union status, race/ethnicity, and first trimester prenatal depressive symptoms (Table 2). Results indicated a significant main effect of the BLOOM intervention, conducted during the second trimester of pregnancy, in predicting pregnancy-related distress measured in the third trimester of pregnancy.

**Table 2.** Multiple Regression of Pregnancy-Related Distress and Depressive Symptoms in the Third Trimester

Variables	Pregnancy-related distress		T3 depressive symptoms	
	β	B (SE)	β	B (SE)
Constant		2.58 (5.52)		−0.95 (5.51)
BLOOM intervention	−0.18^*^	−4.63 (2.17)	−0.02	−0.52 (2.15)
Sociodemographic variables				
Age	−0.13	−0.19 (0.14)	−0.10	−0.18 (0.14)
Education	0.28^**^	1.11 (0.36)	0.17^*^	0.84 (0.36)
Union (living with partner)	0.04	0.58 (1.5)	−0.07	−1.42 (1.5)
Race/ethnicity (White = reference group)				
Black	−0.14	−2.6 (1.81)	−0.03	−0.64 (1.82)
Hispanic	−0.07	−1.6 (2.18)	0.03	0.75 (2.16)
Native	0.05	1.07 (2.11)	0.19^*^	5.26 (2.1)
Depressive symptoms, first trimester	0.38^**^	0.35 (0.08)	0.64^**^	0.72 (0.08)

*n* = 113 with listwise deletion.

^*^ *p* < 0.05; ^**^p < 0.01.

However, the association between the BLOOM intervention and third trimester depressive symptoms was not significant. Education and first trimester depressive symptoms were the only statistically significant control variables across the linear regression analyses.

## Discussion

Findings from the current study indicated that mothers in their second trimester of pregnancy who received the mindfulness-based BLOOM intervention reported significantly lower levels of pregnancy-related distress compared with the control group. These findings provide evidence and highlight the importance of intervention studies that aim at enhancing the mother-infant relationship and improving maternal symptoms of distress, especially during pregnancy.^28,29^

The BLOOM pilot intervention has been previously demonstrated to enhance maternal-fetal bonding during pregnancy,^29^ yet findings from the current study highlight additional psychological benefits, including decreased distress associated with maternal perceptions of pregnancy. It is hypothesized that engaging in these mindfulness-based reflective exercises promotes maternal attentional awareness of their internal mental processes and increased emotional awareness of their relationships with their offspring.

This combination of mentalization and mindfulness appears to be protective, as it reduced stress related to participants' pregnancies. Findings, therefore, highlight promising benefits of using mindfulness-based, reflective interventions to reduce pregnancy-related distress and ultimately promote prenatal well-being.

Notably, the BLOOM intervention had a nonsignificant association with maternal depressive symptoms. Prior studies^22–24^ have found that enhancing maternal-infant bonding decreases perinatal depression, but the samples for those studies only included mothers who had been diagnosed with depression. Our sample was not restricted to those who were depressed, and further, the average depressive symptom score for the sample was below the established cut-off for the CES-D indicating “at risk for depression,” which could explain why we did not find a decrease in depressive symptoms after the intervention.

In addition, although mindfulness-based interventions have been shown to be efficacious for reducing psychological distress, including depression during the perinatal period,^12^ the BLOOM intervention specifically focused on promoting maternal attentional and emotional awareness on the mother-fetal relationship, rather than focusing on mood, or global stress nonspecific to pregnancy.^36^

Further, the intervention was very brief, at 5-minutes per day, which may not be sufficient in terms of the “dose” of the intervention to improve mental health symptoms.^37^ Therefore, including additional reflective exercises that extend beyond the maternal-fetal relationship to address mood and global stress, as well as increasing the amount and/or frequency of the intervention, may be important for promoting maternal mental health more globally.

Finally, the study was limited by the measurement of pregnancy-related distress at only one time point after the intervention. Future studies that include the PDQ at multiple time points are needed to confirm whether the intervention reduces pregnancy-related distress. Despite limitations, however, these findings suggest that a brief, mindfulness-based intervention delivered during pregnancy via cellphone texts is a promising tool to address maternal distress related to pregnancy and promote prenatal well-being.

## Abbreviations Used

BLOOM: Babies and Moms connected by Love, Openness, and Opportunity
CES-D: Center for Epidemiological Studies-Depression
PDQ: Prenatal Distress Questionnaire
